# Hypoxia-induced complement dysregulation is associated with microvascular impairments in mouse tracheal transplants

**DOI:** 10.1186/s12967-020-02305-z

**Published:** 2020-03-31

**Authors:** Mohammad Afzal Khan, Talal Shamma, Shadab Kazmi, Abdullah Altuhami, Hala Abdalrahman Ahmed, Abdullah Mohammed Assiri, Dieter Clemens Broering

**Affiliations:** 1 Organ Transplant Research Section, Comparative Medicine Department, King Faisal Specialist Hospital and Research Centre, Riyadh, Kingdom of Saudi Arabia; 2 Comparative Medicine Department, King Faisal Specialist Hospital and Research Centre, Riyadh, Kingdom of Saudi Arabia; 3 College of Medicine, Alfaisal University, Riyadh, Kingdom of Saudi Arabia; 4 Institute for Research and Medical Consultations, Imam Abdulrahman Bin Faisal University, Dammam, Kingdom of Saudi Arabia

**Keywords:** Complement regulatory proteins, C3d, Acute rejection, Microvascular loss

## Abstract

**Background:**

Complement Regulatory Proteins (CRPs), especially CD55 primarily negate complement factor 3-mediated injuries and maintain tissue homeostasis during complement cascade activation. Complement activation and regulation during alloimmune inflammation contribute to allograft injury and therefore we proposed to investigate a crucial pathological link between vascular expression of CD55, active-C3, T cell immunity and associated microvascular tissue injuries during allograft rejection.

**Methods:**

Balb/c→C57BL/6 allografts were examined for microvascular deposition of CD55, C3d, T cells, and associated tissue microvascular impairments during rejection in mouse orthotopic tracheal transplantation.

**Results:**

Our findings demonstrated that hypoxia-induced early activation of HIF-1α favors a cell-mediated inflammation (CD4^+^, CD8^+^, and associated proinflammatory cytokines, IL-2 and TNF-α), which proportionally triggers the downregulation of CRP-CD55, and thereby augments the uncontrolled release of active-C3, and Caspase-3 deposition on CD31^+^ graft vascular endothelial cells. These molecular changes are pathologically associated with microvascular deterioration (low tissue O_2_ and Blood flow) and subsequent airway epithelial injuries of rejecting allografts as compared to non-rejecting syngrafts.

**Conclusion:**

Together, these findings establish a pathological correlation between complement dysregulation, T cell immunity, and microvascular associated injuries during alloimmune inflammation in transplantation.

## Background

Microvascular loss has been associated with the progression of tissue remodeling and organ rejection during transplantation. Various preclinical and clinical studies have demonstrated a marked loss of microvasculature in nonfibrotic airways a finding suggesting that a loss of microvasculature precedes and may be related to the progression of chronic rejection in transplanted lungs [1–5]. Microvascular loss and associated tissue injuries due to alloimmune inflammation involves an organized communication of myriad of cellular (T cells, B cells, Macrophages, and Dendritic cells) and molecular components (Complement factors and Immunoglobulins) which play a crucial role in the development of tissue remodeling, and progression of small airways closure and subsequent decline in pulmonary functions [1, 2, 6–8]. Microvascular loss during complement-mediated inflammation, and subsequent microangiopathy with its attendant ischemia, can lead to tissue infarction and terminal airway fibrosis [1, 2, 5, 9], and therefore, maintaining healthy microvasculature with various immunotherapies in rejecting allografts may be crucial for preventing terminal airway fibrosis [3, 5, 10].

Role of complement and associated regulatory mechanism has been studied in ischemia/reperfusion injury and other associated pathological alterations during transplantation but a direct pathological impact of C3 activation and regulatory proteins is not well known during alloimmune inflammation post-transplantation. Activation of complement cascade release C3, C5 anaphylatoxins, and other toxic peptides, which play a decisive role in pathophysiological injuries during alloimmune inflammation [11–15]. To shield against the complement-mediated injury, host tissues express several membrane-anchored CRPs, which include decay-accelerating factor (DAF; CD55), membrane cofactor protein (MCP), complement receptor 1-related gene/protein y (Crry), complement receptor 1 (CR1) and CD59 [16, 17]. All these CRPs are unique in targeting and operate only at specific steps of complement activation, which makes them highly sophisticated regulatory switch to protect cellular machinery during the extreme inflammatory phase [17–23].

CD55 inhibits the formation of the C3-convertase, the major amplification step in the complement activation cascade and plays a critical role in negating complement-mediated injury in the pathophysiology of various inflammatory diseases including allograft rejection [24–33]. Here, we proposed to investigate a possible relationship between the key activated complement factor C3, and CD55, which is normally expressed on vascular endothelium and is the focus of the ongoing research plan. Further, we aim to determine a pathological correlation between graft expression of CD55 and C3d during microvascular rejection in an experimental mouse model of orthotopic tracheal transplant [1, 7, 34].

## Methods

### Mice

All MHC-mismatched mice selected for this investigation were originally acquired from the Jackson Laboratory (JAX, USA), and preserved as an original breeder colony in our King Faisal Specialist Hospital and Research Centre animal facility (KFSH&RC) at Riyadh, Saudi Arabia. All animal procedures used in this study were approved by KFSH& RC Animal Care and Use Committee (ACUC).

### BALB/c

BALB/c (H-2d) mice were selected as an allogeneic tracheal graft donor for the C57BL/6 mice recipients in the orthotopic tracheal transplants.

### C57bl/6

C57BL/6 (B6.H-2b) mice were selected as a tracheal graft recipient in all allograft conditions while they were selected as a tracheal graft donor in syngraft conditions as described in the experimental plan (Table 1).

**Table 1:** Details of transplanted groups, and experimental endpoints

Donor	Recipient	Treatment	Group purpose	Assessments (d)
C57BL/6	C57BL/6	Saline	Saline-treated syngraft control	6, 8, 10
BALB/c	C57BL/6	Saline	Saline-treated allograft control	6, 8, 10

Sample size (n) = 6–12 transplants/time points/experiment

### Surgical procedure

The surgical procedure of Orthotopic Tracheal Transplants (OTT) was performed under sterile conditions and different groups were surgically transplanted. In brief, recipient mice were anesthetized (Ketamine 100 mg/kg and Xylazine 20 mg/kg) and trachea was bisected with small incision, and donor tracheal segment of 4–6 rings was surgically sutured into the recipient with 10-0 nylon suture (AROSurgical, USA) at each anterior and posterior end of the bisected trachea. Next, all the surrounding tissue were placed and the overlying skin was closed with 5-0 silk suture [34, 35]. All transplanted mice were given Carprofen (dose 5 mg/kg × SC) and Zolecin (dose 100 mg/kg × SC) and were monitored for any respiratory distress-stridor in the first 24 h after surgery. To examine the rejection and associated microvascular kinetics, allografts were selected at specific days post tracheal transplantation, which starts from d6 (point of early microvascular flow), d8 (point of maximum microvascular flow), d10 (point of microvascular loss/acute rejection) to better see a pathological correlation between expression of active complement factors and CRP-CD55 with associated tissue and microvascular injuries in rejecting allografts [9, 36, 37].

### Graft functional microvasculature

To examine the functional microvascular blood flow between donor and recipient grafts, transplanted mice were anesthetized and quickly injected intravenously with 50 μl (1 mg/ml FITC-conjugated *Lycopersicon esculentum*) of tomato lectin [7] and waited for 5 min before flushing the whole vasculature with 1% PFA (paraformaldehyde) via the aorta. After PFA washing, the graft was harvested and fixed in 1% PFA at 4 °C for 10 min. Next, the graft was mounted and examined by Immunofluorescence microscopy (EVOS FL auto cell imaging system, Life Technologies, USA) to detect the presence of FITC-tagged functional graft microvasculature [9, 37].

### Graft oxygenation and blood flow

The oxygen (tpO_2_ mmHg) and blood perfusion (BPUs) during rejection were measured by combined oxygen and blood flow sensors (model NX-BF/OF/E, Oxford Optronix, UK) as described earlier with some modifications [5, 9, 34, 37]. Briefly, the transplanted mouse was anesthetized and the graft was exposed for oxygen and blood flow measurement. Next, a 23G needle was used to make a hole in the graft, and a combined sensor was gradually inserted through a micromanipulator until it reaches the epithelium of the graft. Since the graft is orthotopic so it always remains in the vicinity of inhaled oxygen, which may interfere with tissue oxygen. To avoid and minimize this inhaled oxygen, the sensor was lowered until the tpO_2_ levels decrease to 5 mmHg or less (indicating a zeroing effect induced by tissue compression), and subsequently raised in small increments through micromanipulator until the tpO_2_ and BPU reading plateaus and a consistent reading was obtained [34, 35]. Of note, detachment of the sensor from the tissue epithelium spiked the oxygen content (> 45 mmHg) therefore we routinely optimized this measurement while the sensor remains in firm contact with epithelium, which we regulate through micromanipulator.

### Immunofluorescence staining

To demonstrate cellular inflammation, and complement dysregulation, we examined harvested allograft at d6 and d10 post-transplantation and immunostained for CD4^+^, CD8^+^ T cells, and vascular endothelial deposition of CD55 and active C3 during post-transplantation. In brief, harvested and Tissue-Tek O.C.T. (Sakura Finetek, Japan) frozen grafts were sliced (5 μm) through a cryostat (HM550; Microm) and the sections were mounted on super frost/plus slides (Fisher Scientific) for immunofluorescence staining as described [3, 5, 9, 37]. After methanol/acetone (1:1) fixation for 10 min at − 20 °C, the slides were washed and incubated with 10% donkey serum for 30 min. Next, slides were overnight incubated with either Rat anti-mouse CD4 (BD biosciences, USA) Rat anti-mouse CD8 (BD biosciences, USA), Rabbit anti-mouse CD55 (Abcam, USA), Goat anti-mouse C3 (R&D systems, USA), Rabbit anti-mouse Caspase-3 (R&D Systems, USA) and Rat anti-mouse CD31 (R&D Systems, USA) specific primary antibodies, and slides were incubated overnight and later rinsed (3×) gently to remove any non-specific binding. Next, slides were incubated with Cy3 donkey anti-rat (Jackson ImmunoResearch Laboratories, USA), Alexa 488 goat anti-rabbit (Jackson ImmunoResearch Laboratories, USA) or with Alexa 488 Donkey anti-goat (Jackson ImmunoResearch Laboratories, USA) secondary antibodies for 1 h. at 37 °C. After incubation, slides were gently rinsed (3×) and mounted in Vectashield mounting medium (Vector Laboratories, USA) for image acquisition. Image acquisition and morphometric analysis of selected markers were performed on randomly selected high-powered fields on EVOS FL auto cell imaging system (Life Technologies, USA), and the percent co-localization of two markers was quantified as mean integrated fluorescence intensity using ImageJ program [7, 9, 37].

### Flow cytometry

To isolate CD55 expressing vascular endothelial cells, collagenase digestion of harvested graft was performed according to the standard protocol with some modifications [38]. Briefly, each freshly harvested graft was minced in cold HEPES buffer, and incubated with 2.5 mg/ml of collagenase D (11088858001 Roche, USA) for 1 h. at room temperature in 50RPM shaking bath. After incubation, the cell suspension was filtered with 70um cell strainers (Falcon, USA), and centrifuged at 400*g* for 30 min. Next, the isolated cell population was stained for surface expression of CD55 on CD31^+^ endothelial cells, and Flow Cytometric analysis was performed at the flow rate of 14 μl/min and a minimum of 500,000 events. All data were later analyzed through BD Accuri C6 integrated software version C6 [5]. Further, to examine the circulating T effector cells (CD4^+^ and CD8^+^) expression during rejection, blood samples were collected (BD-vacutainers) and lymphocyte buffy coat was separated through Hisptopaque gradient procedure as described [3, 5, 39], and mouse T effector specific markers were stained with APC-conjugated anti-mouse CD4^+^ (Clone RM4-5 RUO, BD Pharmingen), and Alexa488-conjugated CD8^+^ (Clone 53-6.7, BD Pharmingen) respectively as recommended by BD Pharmingen assay, which specifically flow sort CD4^+^ and CD8^+^ in a given lymphocytes population. Data were recorded at the flow rate of 14ul/min and a minimum of 500,000 events were collected, and further analyzed through BD Accuri C6 integrated software [3, 5].

### Histopathology

Pathological changes in the airway epithelium of allografts were evaluated by H&E staining as described [3, 5, 40]. In brief, harvested and Tissue-Tek O.C.T. medium (Sakura Finetek, Japan) processed graft sections on super frost/plus slides (Fisher Scientific) were stained by H&E to detect any pathological and structural perturbations in airway epithelium and mononuclear cell infiltration [9, 41].

### Quantitative PCR

RT-PCR analysis of mRNA expression of HIF-1α, CD55, and key inflammatory cytokines was performed with some modifications [3, 5, 9]. Briefly, total RNA from tracheal graft was extracted using RNeasy mini kit 50 (Qiagen Sciences, Maryland, USA.) and quantified using a NanoDrop 1000 spectrophotometer (NanoDrop Technologies, USA). cDNA from each isolated RNA was synthesized with a Superscript™ II cDNA reverse transcription kit (ThermoFisher Scientific) and real time-PCR was performed using gene-specific primers on an AB 7500 Fast Real-Time PCR system in triplicates using Power SYBR Green (ThermoFisher Scientific). Data were analyzed with integrated software, and expression levels were analyzed by the 2^−∆∆Ct^ method after normalization to the housekeeping genes glutaraldehyde dehydrogenase (GAPDH). We selected the Hypoxia-inducible gene (HIF1-α), CD55, and T cell-specific (IL-2 and TNF-α) gene transcripts for the detection of hypoxic, regulatory and inflammatory phases of graft. The sequence of individual primers used in the present study is shown in Table 2.

**Table 2:** A sequence of primers for RT-PCR analysis

Gene	Primer	Sequence
HIF1-α	Forward	ACCTTCATCGGAAACTCCAAAG
	Reverse	CTGTTAGGCTGGGAAAAGTTAGG
IL-2	Forward	GCGGCATGTTCTGGATTTG
	Reverse	TGTGTTGTCAGAGCCCTTTAG
TNF- α	Forward	CCCTCACACTCAGATCATCTTCT
	Reverse	GCTACGACGTGGGCTACAG
CD55	Forward	TAGCCAGGTGGTCACCTATT
	Reverse	GACTGCTCCATTGTCCTACATC
GAPDH	Forward	AACAGCAACTCCCACTCTTC
	Reverse	CCTGTTGCTGTAGCCGTATT

### Statistical analysis

Statistical comparison between groups was performed using two-way ANOVA with post hoc Bonferroni correction for multiple comparisons while single comparisons were analyzed by 1-way ANOVA through GraphPad™ Prism software. A p-value < 0.05 was considered significant.

## Results

### Loss of graft functional microvasculature during rejection

While the deleterious effects of C3 deposition on vascular reestablishment, tissue oxygenation, and blood perfusion had already been reported earlier [9], here we further delineated a fine correlation between CD55/C3d balance, airway microvasculature and epithelial injuries during allograft rejection. As reported in previous transplant settings, activation of the complement cascade and associated complement regulatory protein dysregulation has been a key to affect microvascular blood flow, tissue oxygenation and airway epithelial repair during airway allograft rejection [3, 42]. We hypothesized that vascular inflammation is directly associated with donor-recipient microvasculature, and microvascular flow and tissue oxygenation. In this study, allograft was monitored for tissue oxygenation, microvascular blood perfusion, and the occurrence of donor-recipient microvasculature in the course of airway rejection.

**Fig. 1:**
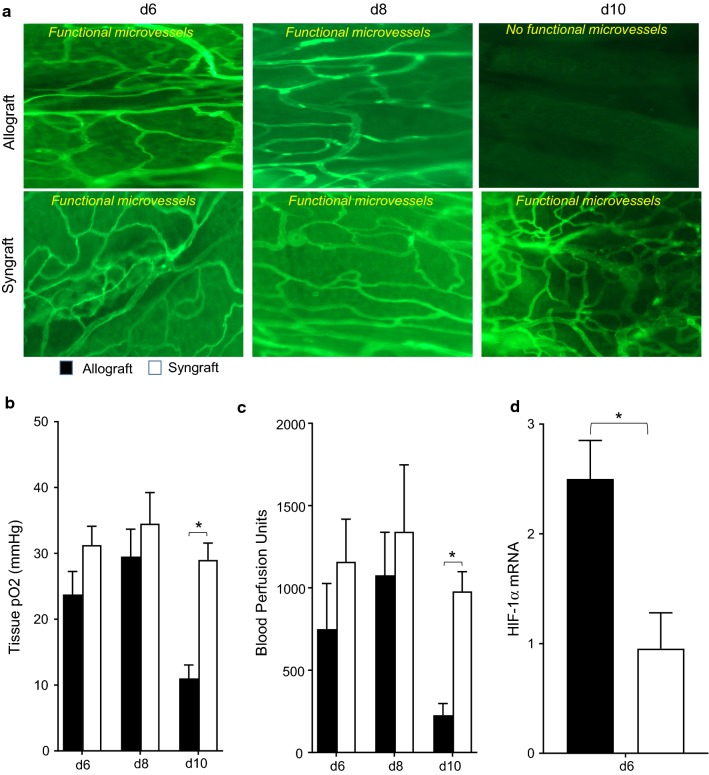
Allograft rejection is associated with donor-recipient microvasculature loss. **a** Lectin binding assay of whole-mount tracheal grafts on d6, d8, and d10. Original magnification, ×20. **b**, **c** Tissue pO_2_ (Mean ± SE, mmHg) and Blood perfusion (Mean ± SE, units) were plotted over different time points. **d** Quantitative RT-PCR analysis shows fold change in HIF1-α mRNA at d6 post-transplantation. Data are presented as means with SE of 6–12 transplants/time point/experiment. *p < 0.05

First, to demonstrate functional microvasculature between the donor and recipient tracheal grafts, we transplanted C57BL/6 recipients with tracheas from MHC-incompatible BALB/c donors (allografts), and harvested trachea at selected time point post-transplantation. To check the microvasculature, rejecting allografts were examined by FITC-lectin binding assay, which specifically maps the existence of functional microvasculature between donor and recipient grafts [9, 31, 37]. We found that allograft remains perfused from d6 to d8 but lost microvascular perfusion at d10 and remained unperfused (Fig. 1a–c). Next, we examined the levels of tissue oxygenation and microvascular blood flow (measured in blood perfusion units, BPUs) in rejecting allografts and in syngraft at d6, d8, and d10 post-transplantation. Our results demonstrate that BALB/c→C57BL/6 allografts remain oxygenated from d6–d8 but pass through a period of hypoxia and ischemia on d10 post-transplantation while syngraft remains oxygenated during this phase (Fig. 1a–c). Next, we examined harvested grafts for HIF-1α mRNA expression during rejection in rejecting allografts. We found a significant early increase in HIF-1α mRNA expression in allograft compared to syngraft controls, which further established a pathological correlation that microvascular injury coincides with the onset of the hypoxic state which eventually triggers higher HIF-1α expression in allograft compared to their control syngraft (Fig. 1d).

### Microvascular loss is associated with cellular inflammation

**Fig. 2:**
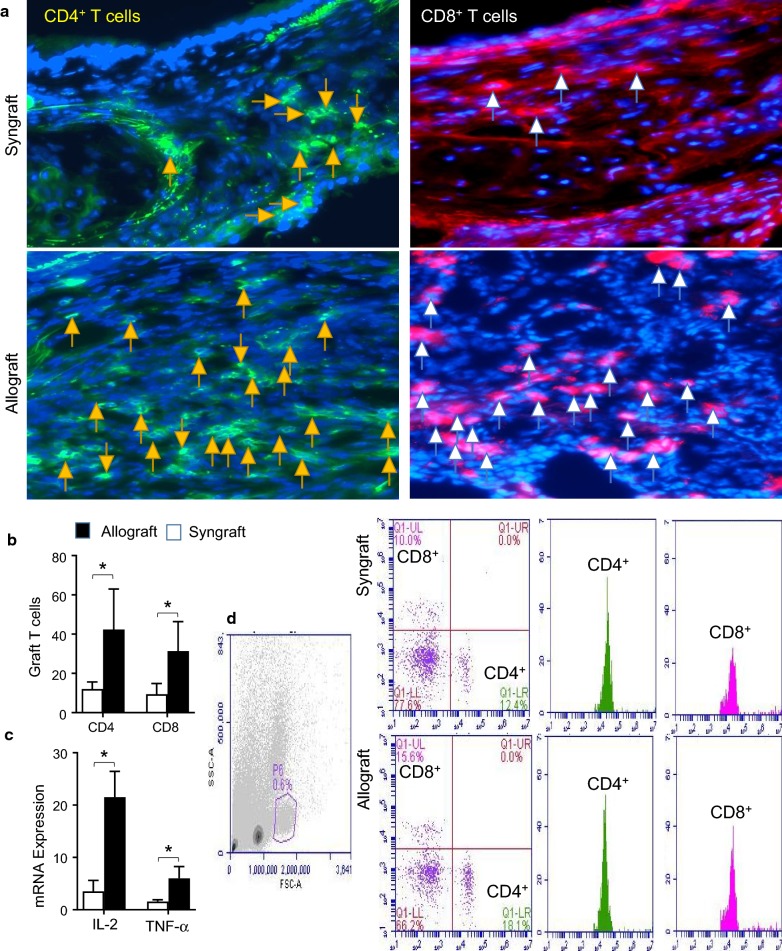
Microvascular loss is associated with an increase in graft T cells infiltration: **a**, **b** Immunofluorescent staining for CD4^+^, and CD8^+^ at 10 post-transplantation. **c** Quantitative RT-PCR analysis shows fold change in IL-2 and TNF-α mRNA at d10 post-transplantation. **d** Flow cytometry analysis of peripheral lymphocytes (CD4^+^, and CD8^+^) at d10 post-transplantation. Data are presented as means with SE of 6–12 transplants/time point/experiment. *p < 0.05

Here, we tested the alloimmune response of peripheral and graft infiltration of CD4^+^ and CD8^+^ T cells during the phase of acute rejection, which is defined by the loss of microvasculature and subsequent hypoxic state. To investigate the inflammatory state during microvascular loss, we surgically transplanted MHC-incompatible C57BL/6 (B6, H-2^b^) with tracheas of BALB/c (H-2^d^) donors. Next, blood lymphocytes were isolated from BALB/c (H-2^d^)→C57BL/6 (H-2^b^) control allografts and C57BL/6 (H-2^b^)→C57BL/6 (H-2^b^) syngraft for peripheral CD4^+^, CD8^+^ T effector cells at d6 and d10 post-transplantation. We found that allografts exhibited a significant increase in CD4^+^ and CD8^+^ T cells both in peripheral blood and graft, compared to syngraft control at d6 and d10 post-transplantation (Fig. 2a, b). These findings indicate that functional microvascular loss during rejection is associated with the increase in both peripheral and graft infiltration of CD4^+^ and CD8^+^ T cells. Collectively, this creates a favorable inflammatory environment to initiate and augment microvascular associated injuries, which play a crucial role in the development of airway tissue remodeling during transplantation. To further confirm the inflammatory state, we performed RT-PCR analysis of two key inflammatory cytokines (IL-2 and TNF-α) in harvested grafts at d10 post-transplantation (Fig. 1c). Our mRNA expression analysis demonstrated that T cell-specific cellular infiltration is associated with an influx of proinflammatory cytokines, which will play a decisive role in tissue microvascular injury during immune rejection.

### Microvascular loss is associated with complement dysregulation

To avoid complement-mediated injury, host tissues express a number of membrane-anchored CRPs including decay-accelerating factor (DAF; CD55). To investigate the complement dysregulation and potential complement-mediated graft microvascular injuries during transplantation, tracheal allografts (BALB/c→B6) were examined through mRNA expression, flow cytometry and immunofluorescence imaging at d6, and d10 post-transplantation.

**Fig. 3:**
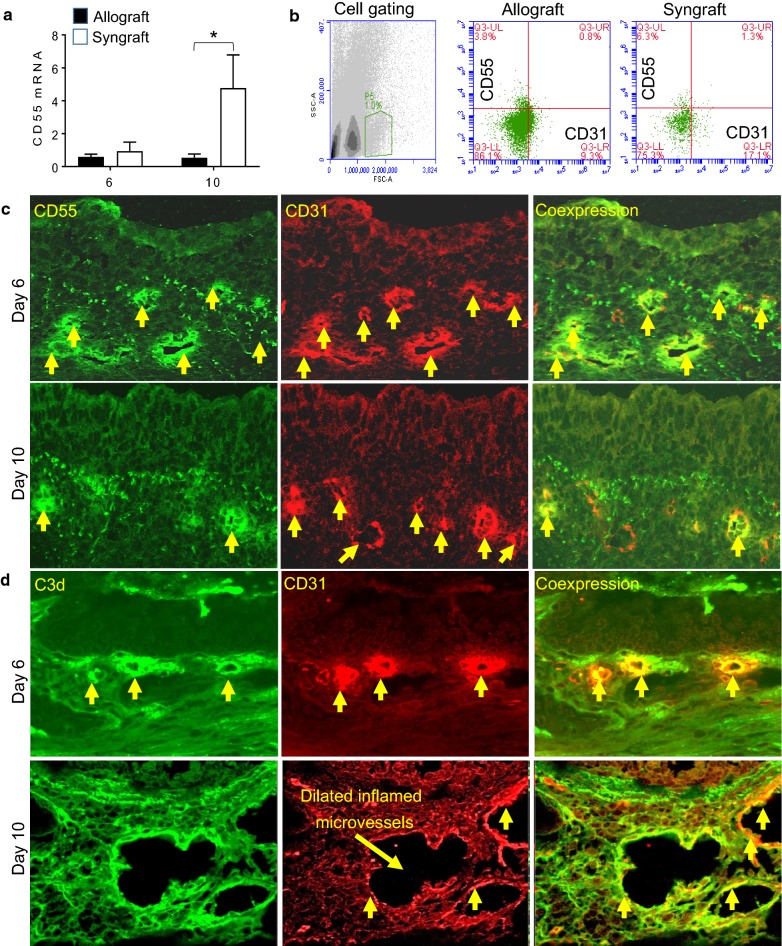
Microvascular loss is associated with complement dysregulation on vascular endothelial cells. **a** Quantitative RT-PCR analysis shows fold change in CD55 mRNA at d6 and d10 post-transplantation. **b** Flow cytometry analysis of vascular endothelial cells isolated from collagenase digested allograft and syngraft at d10 post-transplantation. **c** Immunofluorescent staining for CD55, and CD31 at d6, and 10 post-transplantation in rejecting allografts. **d** Immunofluorescent staining for C3d, and CD31 at d6, and 10 post-transplantation in rejecting allografts. Data are presented as means with SE of 6–12 transplants/time point/experiment. *p < 0.05. Original magnification, ×40

To evaluate the downregulation of CD55 on graft microvascular stratum, we first harvested allografts and syngrafts at d6 and d10 post-transplantation and tested the mRNA expression of CD55. The pattern of mRNA expression showed a significant low CD55 mRNA expression in both d6 and d10 allografts as compared to the corresponding syngraft. To further confirm the CD55 expression during rejection, we harvested both allograft and syngraft and processed them for collagenase digestion assay. The liberated vascular endothelial cells after collagenase treatment were filtered and immunostained for surface expression of CD55 and CD31 proteins. The acquired immunostained cells through flow cytometry exhibited a lower expression of CD55 on CD31^+^ vascular endothelial cells at d10 post-transplantation in allografts as compared to syngraft controls. In addition, both allograft and syngraft were further examined through immunofluorescence staining at d10 (point of microvascular loss) post-transplantation. Image analysis of CD55 and CD31 coexpression further revealed a downward trend in CD55 expression on CD31^+^ vascular endothelial cells at d6 and d10 post-transplantation. These findings suggest that vascular endothelial expression of CD55 down-regulated during allograft rejection at d10 post-transplantation which coincides with the loss of microvasculature (Fig. 3a–c).

To investigate the correlation of CD55 downregulation on associated complement activation, we next examined the deposition of activated complement factor C3d on vascular endothelial cells at d6 and d10 post-transplantation. Our immunofluorescence data demonstrated an upward trend in C3d deposition on CD31^+^ vascular endothelial cells at d6-d10 post-transplantation. These findings suggested that vascular endothelial deposition of active C3 is a major molecular signature of tissue microvascular injury during rejection. These findings support the notion that d10, which is the point of microvascular loss coincides with the low deposition of CD55 and high deposition of C3d on vascular endothelial cells as seen in rejecting allograft compare to syngrafts (Fig. 3a–d). Of note, the syngrafts do not show any significant increase in both CD55 and C3d expression on CD31^+^ vascular endothelial cells.

### Microvascular loss is associated with endothelial caspase-3 activation

**Fig. 4:**
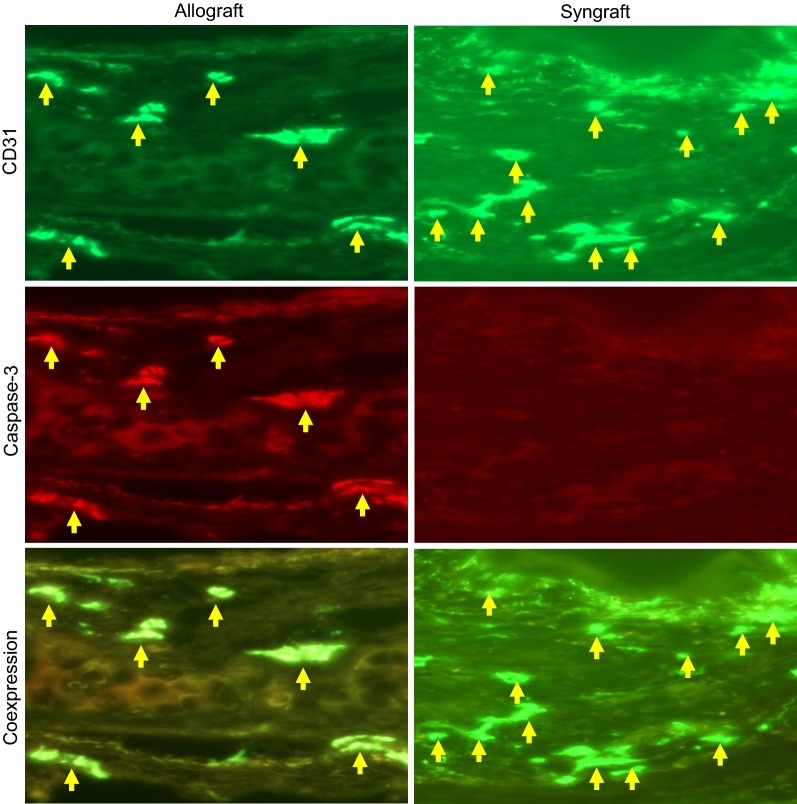
Microvascular loss is associated with Caspase-3 activation on vascular endothelial cells. Immunofluorescent staining for Caspase-3 and CD31 at d10 post-transplantation. Data are presented as means of 12 transplants/time point/experiment. *p < 0.05. Original magnification, ×40

Allografts endothelial cells are a primary target of vascular injury during cytotoxic T lymphocytes (CTL) mediated alloreactivity, which greatly impacts microvascular health and progressive vascular loss. To determine the vascular expression of CTL mediated caspase-3 activation, harvested allograft, and syngraft sections were immunostained for Caspase-3 deposition on vascular endothelial cells at d10 post-transplantation, which coincides with the CD8^+^ T cells infiltration in the graft. Immunofluorescence staining demonstrated a significant increase in Caspase-3 on CD31^+^ vascular endothelial cells during inflammation. These findings suggested that vascular endothelial deposition of active Caspase-3 is a key apoptotic factor generated due to CTL mediated alloreactivity during microvascular injury (Fig. 4).

### Microvascular loss is associated with airway epithelial injury

**Fig. 5:**
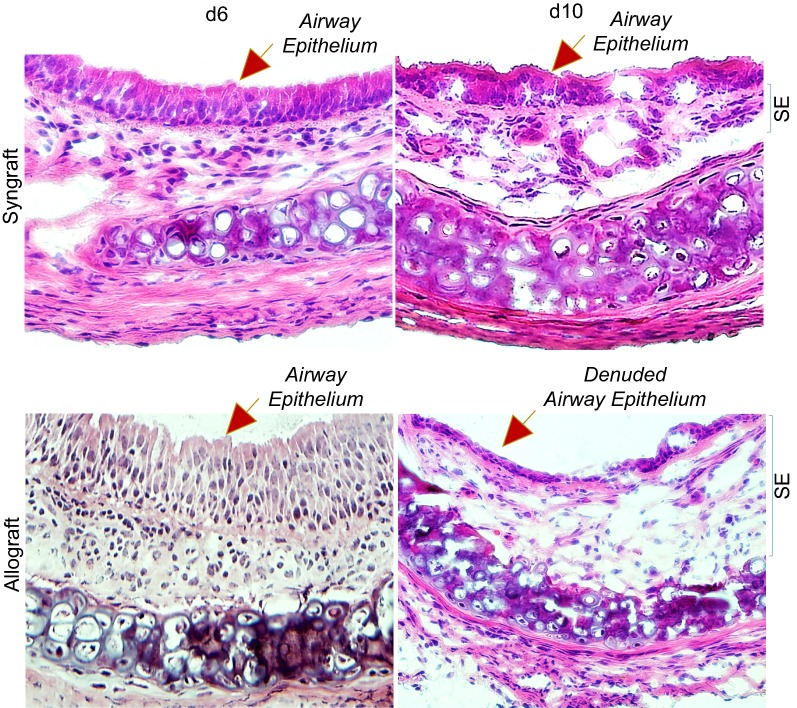
Microvascular loss is associated with an epithelial injury. H&E staining of BALB/c→C57BL/6 allografts at d6, and d10 post-transplantation. SE designate graft sub-epithelial area. Data are presented as means of 12 transplants/time point/experiment. *p < 0.05. Original magnification, ×40

Airway epithelial injuries are the leading pathological event, and key irreversible pathological consequences that proceed when rejecting allografts undergo a state of severe hypoxia/ischemia, and ultimately to airway remodeling during the terminal phase of rejection [37]. To examine airway epithelial injuries, slides were stained by H&E to detect airway epithelial structures in allografts and syngrafts at d6 and d10 post-transplantation as described previously [40] and stained graft slices were imaged using a Leica light microscope to localize epithelial and subepithelial zones. Microscopic examinations of H&E showed an inflamed airway epithelium with massive infiltration of mononuclear cells in subepithelial tissues at d6 post-transplantation, while d10 allograft showed a partial or complete loss of airway epithelium but subepithelial tissues remain populated with massive mononuclear cell infiltration [9, 41]. However, corresponding syngrafts showed no sign of airway epithelial injury or infiltration of subepithelial mononuclear cells during transplantation (Fig. 5).

## Discussion

CRPs are the crucial regulatory switch available to check the uncontrolled active complement deposition and negate their toxic effects on graft vasculature during alloimmune reactions [43, 44]. CRPs, including CD55, complement receptor 1-related gene/protein y (Crry) and CD59 usually modulate deleterious effects of active complement mediators during allograft rejection [21, 28]. Complement accumulation on the vascular endothelium precedes the development of airway fibrosis and subsequent destruction of blood vessels and microvascular blood flow to the rejecting allograft. In addition, previous findings have strengthened the fact that complement deficiency or blockade leads to markedly improved oxygenation in rejecting allografts [5, 9, 37]. To date, work focusing on the impact of hypoxia on CRP expression has been limited to in vitro studies [45, 46]. While these studies have been important in showing how CRPs can be upregulated in cultured cells in hypoxic conditions, they cannot replicate the complex in vivo milieu of a rejecting transplant [47]. Inverse correlation of tissue expression of active complement components and CRPs have been crucial to the progress of transplant injury and associated tissue remodeling during rejection [48–50].

The key hypothesis to be tested in this manuscript is that during alloimmune inflammation, graft microvasculature and the airway epithelium is rendered susceptible to complement-mediated injury through decreased expression of major complement regulatory protein CD55 during a mouse model of OTT. The OTT model is a well-established model to investigate the complement-mediated graft ischemia and airway tissue remodeling during rejection [5, 9, 37]. This study was, therefore, the first to examine the vascular expression of CD55, C3d kinetics, and T cell immunity, and how it correlated to the development of microvascular loss and airway tissue remodeling.

Herein, we demonstrated that early hypoxic state favors a cell-mediated inflammation, which proportionally triggers the downregulation of CD55, and thereby augments the uncontrolled release of active-C3, and Caspase-3 deposition on graft vascular endothelial cells. As reported in previous preclinical studies, alloimmune inflammation is characterized by a massive T cell infiltration (CD4^+^ and CD8^+^), associated proinflammatory cytokines, vascular deposition of CTL mediated Caspase-3 activation, which mutually take part in tissue and microvascular damage [9, 15, 37]. Our preliminary results also demonstrated a significant increase in early activation of HIF-1α mRNA (d6), followed by a massive increase in T cell infiltration and major proinflammatory cytokines (IL-2 and TNF-α) during microvascular deterioration. These findings supported a notion that inflammation-mediated microvascular injury determines hypoxia/ischemia state and consequently led to the early activation of HIF-1α which plays a vital role in the metabolic switches that favor cellular adaptation to hypoxic state [51–53]. To further dissect the impact of HIF-1α on complement regulatory protein CD55 on vascular endothelial cells, we examined rejecting allografts by mRNA expression, immunofluorescence, and flow cytometry experiments at d10 (point of acute rejection) to confirm if the vascular expression of CD55 and activated complement C3 corresponds to microvascular associated injuries during alloimmune inflammation. We observed that complement-mediated alloimmune inflammation and associated regulatory machinery (e.g. CD55) showed an inverse pathological relationship during airway inflammation. Furthermore, we found that vascular endothelial expression of C3d increases, while vascular endothelial expression of CD55 shows a corresponding decrease during allograft rejection. Next, we further evaluated the microvascular state of graft during the downregulation of CD55/upregulation of C3d. Our results indicate that C3d mediated alloimmune inflammation and corresponding downregulation of CD55 showed a strong pathological correlation with alloimmune inflammation (CD4^+^ and CD8^+^), and associated tissue microvascular injuries, graft hypoxia, ischemia, and airway epithelial loss during airway rejection. Altogether, this unfavorable environment seems to contribute to a proinflammatory and more vascular disruption during an alloimmune inflammation and may help to establish a state of severe hypoxia and ischemia. This hypoxic state triggers an inflammatory response, which follows the recruitment of immune cells and associated proinflammatory cytokines. The role of hypoxia has been extensively reported in various immunological conditions including transplantation, and the occurrence of hypoxia has been narrated as a key phase that affects inflammation during the molecular, and cellular checkpoints [54, 55]. It has been well reported that HIF-1α, as the master regulator of oxygen homeostasis, is an important modulator of the tissue repair phase through its signaling in cell migration, cell survival, cell division, growth factor release, and matrix synthesis during the repair [56, 57]. In addition, hypoxia results in tissue ischemia and the expression of a pro-inflammatory state through the downregulation of complement regulatory protein, CD55 [50]. The crucial role of pathological remodeling during persistent inflammation in small airways has been reported in both preclinical and clinical transplantation data, which obstruct the regeneration of airway epithelium and promote fibro-proliferation due to aberrant tissue repair [58–62]. These epithelial injuries have been recognized as a key intermediate step that is ultimately leading to obliterative airway disease [58–61]. As reported earlier, C3 in the first major active fragment, which has been reported to initiate microvascular associated injuries through the graft vascular-endothelial deposition [5, 9, 13, 15, 37, 63]. In addition, endothelial deposition of C3 leads to the onset of vascular leakiness, microvascular congestion, thrombus formation, pulmonary edema and neutrophilic invasion of the microvasculature through the interaction of various inflammatory cells [15, 64]. Complement-mediated vascular injury contributes to the pathophysiology of multiple diseases including ischemia–reperfusion injury, myocardial infarction, and various transplantation conditions [65–67]. Complement has been recognized as a key regulator of adaptive immunity and various activated fragments have been associated with allograft injury through multiple mechanisms, which include post-transplant IR injury, microvascular injury, alloantibody formation, differentiation and function of alloreactive T cells, and contributes to chronic allograft dysfunctions [27, 28, 68–78]. CD55 is a central complement activation regulator that checks the complement activation through the assembly of C3 convertase (C3bBb), and play a key role in various protective and regulatory impacts of CD55 in various transplant models [23, 27, 79–81].

In summary, our results conclude that activation of the complement pathway and corresponding downregulation of CD55 during an alloimmune inflammation are one of the main players in microvascular associated allograft injury. The delineation of this pathological correlation between CRP, activated C3 and tissue remodeling favors the possibility that targeted blocking of activated C3 or the use of recombinant CD55 could be a good therapeutic combination to support microvascular reestablishment during allograft rejection.

## Conclusion

Taken together, these findings highlight the key modulatory effects of complement on microvascular associated injuries, and demonstrate a proof-of-concept that targeting C3 blockade could facilitate CD55-mediated vascular protection to allografts. This comparative analysis collectively emphasized that complement activation during alloimmune inflammation is harmful to transplanted tissue, in part, because they contribute to the progression of hypoxia/ischemia by disconnecting microvascular flow between donor-recipient grafts and thus favor hypoxic state. Further, these findings demonstrated a detailed insight of alloimmune response and a fine pathological correlation between complement dysregulation and associated microvascular injuries during rejection. Altogether, this could provide a pathological tool to understand the ongoing pathological state of rejecting graft, and also to better design an anti-complement therapy aimed at preventing microvascular and epithelial injury during alloimmune inflammation.

## Data Availability

The datasets used and/or analyzed during the current study are available from the corresponding author on request.

## Abbreviations

BPU: Blood perfusion units
BOS: Bronchiolitis obliterans syndrome
CRP: Complement regulatory proteins
D: Day
DAF (CD55): Decay *accelerating factor*
MCP: Membrane cofactor protein
CTL: Cytotoxic T lymphocyte
Crry: Complement receptor 1-related gene/protein y
CR1: Complement receptor 1
OTT: Orthotopic tracheal transplant model
PFA: Paraformaldehyde
Tregs: Regulatory T cells
tO_2_: Tissue oxygen
O.C.T: Optimal cutting temperature
